# Lifetime risk of being diagnosed with, or dying from, prostate cancer by major ethnic group in England 2008–2010

**DOI:** 10.1186/s12916-015-0405-5

**Published:** 2015-07-30

**Authors:** Therese Lloyd, Luke Hounsome, Anita Mehay, Sarah Mee, Julia Verne, Alison Cooper

**Affiliations:** Evidence Team, Prostate Cancer UK, 4th Floor Counting House, 53 Tooley Street, London, UK; Knowledge and Intelligence Team (South West), Public Health England, 2 Rivergate, Temple Quay, Bristol, UK

**Keywords:** Asian, Black, Epidemiology, Ethnicity, Lifetime risk, Prostate cancer, White

## Abstract

**Background:**

In the UK, a man’s lifetime risk of being diagnosed with prostate cancer is 1 in 8. We calculated both the lifetime risk of being diagnosed with and dying from prostate cancer by major ethnic group.

**Methods:**

Public Health England provided prostate cancer incidence and mortality data for England (2008–2010) by major ethnic group. Ethnicity and mortality data were incomplete, requiring various assumptions and adjustments before lifetime risk was calculated using DevCan (percent, range).

**Results:**

The lifetime risk of being diagnosed with prostate cancer is approximately 1 in 8 (13.3 %, 13.2–15.0 %) for White men, 1 in 4 (29.3 %, 23.5–37.2 %) for Black men, and 1 in 13 (7.9 %, 6.3–10.5 %) for Asian men, whereas that of dying from prostate cancer is approximately 1 in 24 (4.2 %, 4.2–4.7 %) for White men, 1 in 12 (8.7 %, 7.6–10.6 %) for Black men, and 1 in 44 (2.3 %, 1.9–3.0 %) for Asian men.

**Conclusions:**

In England, Black men are at twice the risk of being diagnosed with, and dying from, prostate cancer compared to White men. This is an important message to communicate to Black men. White, Black, and Asian men with a prostate cancer diagnosis are all as likely to die from the disease, independent of their ethnicity. Nonetheless, proportionally more Black men are dying from prostate cancer in England.

**Electronic supplementary material:**

The online version of this article (doi:10.1186/s12916-015-0405-5) contains supplementary material, which is available to authorized users.

## Background

Prostate cancer is the most common cancer in men in the UK, with 41,736 cases diagnosed in 2011 [1]. Incidence rates have risen over the last 25 years, largely attributable to the introduction of prostate-specific antigen (PSA) testing [1], and prostate cancer is predicted to become the most commonly diagnosed cancer overall in the UK by 2030 [2]. Some cases of prostate cancer grow so slowly that they might never present any symptoms during the man’s lifetime and might therefore never be clinically diagnosed [3]. Thus, incidence of prostate cancer captures not only biological predisposition but also health-seeking behaviour and access to diagnostic testing. Prostate cancer is the second most common cause of cancer deaths in men in the UK, after lung cancer [4–6], with 10,837 deaths recorded in 2012 [1].

Risk factors for prostate cancer include increasing age [7], a family history of the disease in a first-degree relative [8–10], body weight [11], and ethnicity [12]. Prostate cancer incidence rate data in England show that Black (Black African, Black Caribbean, and Other Black) men are significantly more likely, and Asian (Indian, Pakistani, Bangladeshi, and Other Asian) men significantly less likely, to be diagnosed with the disease compared to White men [13]. The Prostate Cancer in Ethnic Subgroups (PROCESS) study [14], and others [15, 16], calculated Black men are 2 to 3 times more likely to be diagnosed with prostate cancer compared to White men of the same age in the UK. In addition, the PROCESS study showed Black men may be diagnosed 5 years younger than White men [17, 18], despite equal access to diagnostic services between ethnic groups [18].

A systematic review and meta-analysis by Evans et al. [19] showed that Black men diagnosed with prostate cancer have a poorer prognosis compared to White men. However, all of the studies included in the analysis were based in the United States, where the poorer prognosis in Black men is thought to be due to their less privileged socioeconomic position, and therefore reduced access to health services which require the patient to pay, and not necessarily due to them being more likely to be diagnosed with aggressive prostate cancer [19]. Analysis of the UK PROCESS study found no evidence of a difference in disease characteristics (stage and Gleason score) at the time of prostate cancer diagnosis or of under-investigation or under-treatment in Black men compared with White men of the same age in the UK [20]. Black men are more likely to undergo radical treatment compared to White men, although this can be largely explained by their younger age at diagnosis [16, 20, 21]. Prostate cancer survival data in the UK show no significant difference in survival rates between Black and White men [13, 16, 22]. However, the high proportion of cases with unknown ethnicity makes interpretation of these results extremely difficult. Increasing survival and an aging population have led to more men dying from prostate cancer at an older age. Prostate cancer mortality rates have been calculated as being 30 % higher in Black than in White men in England, although this was not completely adjusted for population age, so it is difficult to draw conclusions on differences in mortality between ethnic groups [23].

The reasons for the increased risk of prostate cancer in Black men are not yet fully understood, partly due to the exclusion or under-representation of Black men in large-scale genome wide association studies, such as that conducted by Eeles et al. [24], and clinical trials such as the European Randomized Study of Screening for Prostate Cancer and the Prostate, Lung, Colorectal, and Ovarian Cancer Screening trial [25].

It is currently estimated that 1 in 8 men in the UK will be diagnosed with prostate cancer at some point during their lives (lifetime risk of diagnosis) [26]. No estimate for the lifetime risk of dying from prostate cancer has been calculated. Neither the lifetime risk of diagnosis nor dying are known by ethnic group. The PROCESS study [14], and others [15, 16], showed Black men are 2 to 3 times more likely to be diagnosed with prostate cancer compared to White men of the same age in the UK (relative risk). However, this relative risk figure does not provide an individual with information on his personal chance of being diagnosed with prostate cancer or with the information required in order to make an informed decision about whether or not to have a PSA test. Therefore, the aim of the present study was to calculate both the lifetime risk of being diagnosed with and dying from prostate cancer by major ethnic group in England in order to provide updated and improved information on the impact of ethnicity on prostate cancer risk and to raise awareness amongst those ethnic groups at higher than average risk.

## Methods

### Calculating lifetime risk

‘Lifetime risk’ is an estimation of the risk that a newborn child has of a certain event occurring at some point during their life. Lifetime risk calculations are based on current incidence and mortality rates and are therefore calculated under the assumption that the current rates, within each age group, will remain constant during the life of the newborn child. Lifetime risk is usually expressed as a percentage, e.g., 20 %, or using odds, e.g., 1 in 5. Lifetime risk odds are rounded up to avoid overestimating risk, e.g., 1 in 4.1 would be rounded up to 1 in 5.

There are several possible methods to calculate the lifetime risk of being diagnosed with and of dying from prostate cancer, but the most appropriate is the ‘competing risks’ methodology. All results herein were calculated using the analytical program package DevCan version 6.7.0, which uses this methodology [27–29] and is similar to the ‘Current Probability’ method [30] used by others for prostate cancer risk [31]. Both these methods are appropriate when it is rare to have more than one diagnosis of the same cancer over the course of a lifetime, as with prostate cancer [32]. Both methods create hypothetical life tables. However, DevCan more comprehensively accounts for competing risks by calculating the number of men who are alive and disease-free in each 5 year age band, as opposed to just the total number alive. In addition to calculating the residual lifetime risk in each age group, DevCan also enables the calculation of confidence intervals (CIs).

### Ethnic group classifications

Two classifications of a person’s ethnic group are used in censuses and other records, including Hospital Episode Statistics (HES); these are major ethnic groups (‘White’, ‘Black’, ‘Asian’, ‘Mixed’ and ‘Other’) and, within each of these, minor ethnic groups. This study presents the lifetime risk of diagnosis and dying from prostate cancer in the major ethnic groups ‘White’, ‘Black’, and ‘Asian’ only. This study has not calculated lifetime risk within the minor ethnic groups due to the small number of prostate cancer incident cases and deaths in these groups. The major ethnic group ‘White’ is made up of ‘White British’, ‘White Irish’, and ‘Other White’; the major ethnic group ‘Black’ is made up of ‘Black African’, ‘Black Caribbean’, and ‘Other Black’; and the major ethnic group ‘Asian’ is made up of ‘Indian’, ‘Pakistani’, ‘Bangladeshi’, and ‘Other Asian’. Table 1 shows the average male population by major and minor ethnic groups in England for 2008–2010. The major ethnic groups ‘Mixed’ (made up of ‘White & Black Caribbean’, ‘White & Black African’, ‘White & Asian’, and ‘Other Mixed’) and ‘Other’ (made up of ‘Chinese’ and ‘Other’) were not analyzed in this study as the constituent minor ethnic groups cover a wide range of mixed ethnicities, making it difficult to attribute any potential differences in lifetime risk to one particular ethnicity.

**Table 1:** Estimated average male population by major and minor ethnic group, England 2008–2010

Major ethnic group	Estimated average annual male population 2008–2010 ^a,b^, N (% of total population)		Minor ethnic group ^a,b^, (% of the major ethnic group)	
White	22,199,289	(87)	White British	(94)
			White Irish	(1)
			Other White	(4)
Asian	1,697,627	(7)	Indian	(42)
			Pakistani	(31)
			Bangladeshi	(12)
			Other Asian	(16)
Black	790,462	(3)	Black Caribbean	(36)
			Black African	(53)
			Other Black	(11)
Mixed	511,721	(2)	White & Black Caribbean	(33)
			White & Black African	(13)
			White & Asian	(30)
			Other Mixed	(24)
Other	436,551	(2)	Chinese	(47)
			Other	(53)
Total	25,635,649			

^a^ The populations by ethnic group for 2008 and 2009 are estimated by the Office for National Statistics (ONS) using experimental statistics but should be viewed with caution as they have not been shown to meet the standards required of National Statistics

^b^ The population by ethnic group for 2010 was estimated using the 2011 Census data (with minor ethnic group Chinese reclassified under major group Other for consistency with other data sources)

Table produced with data from [33–35]

### Availability and access to data

A lack of data on ethnicity created difficulty in calculating the lifetime risk of diagnosis and dying by major ethnic group. Prostate cancer incidence is recorded by cancer registries and, depending on the data source, information on ethnicity is variable. The Office for National Statistics (ONS) also holds incidence data but with no information on ethnicity. The ONS is the official source of data on mortality, but these data do not include ethnicity as it is not recorded on death certificates.

Additional file 1 shows the data sources used to gather data on prostate cancer incidence, prostate cancer mortality, all-cause mortality, and population estimates, available where possible by ethnic group, which were then combined in order to produce the final datasets (shown in grey boxes) required to calculate the lifetime risk of being diagnosed with, and dying from, prostate cancer by major ethnic group using the DevCan software. More details on the data sources in Additional file 1 are outlined below.

### Incidence and mortality data

Public Health England (PHE) run cancer registration in England and created the 1990–2010 England National Cancer Data Repository (NCDR) Analysis Dataset, which brought together data from each English Cancer Registry for the period 1990–2010. In accordance with the National Health Service (NHS) Act 2006, PHE is permitted to hold and process cancer data on people without their explicit consent. NCDR data on men diagnosed with prostate cancer were linked to the HES database, which contains data on inpatient and day case episodes for patients. The HES records contain a self-reported ethnicity field and so this database is the main source of ethnicity data for cancer patients. Linkage between NCDR and HES was based on NHS number, or postcode and date of birth if NHS number was not available. Overall, 99 % of people diagnosed with cancer were able to be linked.

PHE also holds a pseudonymised database of ONS death registration data linked to HES, created by the Health and Social Care Information Centre (‘HSCIC HES-ONS linked mortality data’; Additional file 1) [36]. This database allowed an ethnicity to be assigned to a death record in the same way as previously described. Prostate cancer deaths were identified by the documented underlying cause of death, which is defined by the World Health Organization, in accordance with the rules of the International Classification of Diseases, as “the disease or injury that initiated the train of morbid events directly leading to death, or the circumstances of the accident or violence which produced the fatal injury” [37]. This ensures that the prostate cancer mortality dataset only contains men who died from prostate cancer and not simply with it.

PHE provided prostate cancer incidence, prostate cancer mortality, and all-cause mortality by 5-year age groups and major ethnic groups for 2008, 2009 and 2010 in England (Additional file 1). As these were aggregated figures from routinely collected data, no ethical approval was needed for this study.

### Population data from the Census

Census data, which are the most accurate source of population data on ethnicity in England, are only collected every 10 years (the latest Census being a snapshot of 27^th^ March 2011). ONS released mid-year population estimates by ethnic group and 5-year age groups for each year up to 2009, but these are “experimental” statistics which have not received formal National Statistics status [38]. However, together with the 2001 and 2011 Censuses, these are the only population data by ethnicity and 5-year age groups available for this period. We therefore used the 2011 Census data as an approximation of the 2010 population, and the experimental mid-year population estimates for 2008 and 2009 (Additional file 1), to calculate the average male population by major ethnic group in England for 2008–2010 (Table 2). In the 2011 Census, the minor ethnic group ‘Chinese’, which had in previous records been included under the major ethnic group ‘Other’, was now included under the major ethnic group ‘Asian’. In this current study, to maintain consistency with the mid-year estimates and HES, the minor ethnic group ‘Chinese’ was re-coded to be included under the major ethnic group ‘Other’.

**Table 2:** Male population estimates by major ethnic group, by individual years and averaged, England 2008–2010

Major ethnic group	2008 ^a^, N (%)		2009 ^a^, N (%)		2010 ^b^, N (%)		Average annual population (2008–2010) ^a,b^, N (%)	
White	22,165,900	(88)	22,233,900	(87)	22,198,066	(85)	22,199,289	(87)
Asian	1,564,400	(6)	1,623,800	(6)	1,904,680	(7)	1,697,627	(7)
Black	731,800	(3)	751,400	(3)	888,185	(3)	790,462	(3)
Mixed	458,300	(2)	481,200	(2)	595,664	(2)	511,721	(2)
Other	403,000	(2)	424,100	(2)	482,553	(2)	436,551	(2)
Overall	25,323,400		25,514,400		26,069,148		25,635,649	

^a^ The populations by ethnic group for 2008 and 2009 are estimated by the Office of National Statistics (ONS) using experimental statistics but should be viewed with caution as they have not been shown to meet the standards required of National Statistics

^b^ The population by ethnic group for 2010 was estimated using the 2011 Census data (with minor ethnic group Chinese reclassified under major group Other, for consistency with other data sources)

Table produced with data from [33–35]

### Discrepancies between PHE-supplied and ONS data

When comparing the number of deaths, discrepancies were found between PHE-supplied data and the original ONS data. Between 2008 and 2010, there were 112,734 all-cause deaths recorded in ONS data that did not appear in PHE-supplied data (671,567 (ONS) vs 558,833 (PHE-supplied; Table 3). Of these missing deaths, 2,158 were recorded as due to prostate cancer (in addition to the 24,363 prostate cancer deaths recorded in PHE-supplied data); the exact reasons for this discrepancy were unclear. The ONS dataset remained the ‘gold standard’ reference for overall number of all-cause and prostate cancer-specific deaths (for all ethnicities combined).

**Table 3:** Prostate cancer incident cases/deaths and all-cause deaths by major ethnic group and methodology, England 2008–2010

	Major ethnic group	Data source	Raw data	Method for assigning ethnicity to unknown cases		
				All White	Proportionate	Increased minority
Number of men diagnosed with prostate cancer, n	White	NCDR	71,620	97,974	**96**,**489**	95,746
	Asian	NCDR	1,097	1,097	**1**,**478**	1,668
	Black	NCDR	2,402	2,402	**3**,**236**	3,653
	Unknown	NCDR	26,354			
	Overall	NCDR	102,252			
		ONS	100,378			
Number of men who died from prostate cancer, n	White	PHE	22,238	23,354	23,306	23,281
		PHE/ONS ^a^		25,512	**25**,**370**	25,299
	Asian	PHE	227	227	238	243
		PHE/ONS ^a^			**259**	275
	Black	PHE	584	584	612	626
		PHE/ONS ^a^			**666**	707
	Unknown	PHE	1,116			
		PHE/ONS ^a^	3,274			
	Overall	PHE	24,363			
		ONS	26,521			
Number of men who died from any cause, n	White	PHE	498,471	533,310	531,613	530,765
		PHE/ONS ^a^		646,044	**638**,**856**	635,262
	Asian	PHE	11,935	11,935	12,729	13,125
		PHE/ONS ^a^			**15**,**296**	16,977
	Black	PHE	7,202	7,202	7,681	7,920
		PHE/ONS ^a^			**9**,**230**	10,244
	Unknown	PHE	34,839			
		PHE/ONS ^a^	147,573			
	Overall	PHE	558,833			
		ONS	671,567			

NCDR, National Cancer Data Repository; ONS, Office for National Statistics; PHE, Public Health England

^a^ PHE data with additional ONS mortality counts

Numbers used for ‘best estimate’ lifetime risk are highlighted in bold

Note: numbers do not add up as major ethnic groups Mixed and Other are not included

Table produced with data from [39–43]

There was also a discrepancy in prostate cancer incidence between NCDR and ONS data. Unlike the mortality data, the number of incident cases of prostate cancer was higher in NCDR data than in ONS data for the period 2008–2010 (Table 3). This discrepancy originates from the 2008 and 2009 data (2008: 32,186 (NCDR) vs 30,893 (ONS); 2009: 35,243 (NCDR) vs 34,593 (ONS)). This difference is primarily accounted for by ONS data being fixed at the time of publication and therefore only including registrations collected up to a certain point, whereas cancer registrations continue to be added in retrospect after the end of the year. This is likely to explain why the difference in numbers was smaller in 2009, compared to 2008, and the 2010 data were very closely matched (2010: 34,823 (NCDR) vs 34,892 (ONS)). NCDR data were considered more complete and therefore the gold standard for the number of prostate cancer incident cases in this study.

When using the different sources of data to calculate the lifetime risk of being diagnosed with prostate cancer in England, for all ethnicities combined, the effect of these data discrepancies can be seen (Table 4). There was a difference in the lifetime risk of diagnosis when using NCDR incidence data (14.8 %; 95 % CI, 14.7–14.9 %, or 1 in 7) compared to ONS incidence data (13.1 %; 95 % CI, 13.0–13.2 %, or 1 in 8). The ONS data result corresponded more closely to the widely used Cancer Research UK statistic for the UK (13.2 % or 1 in 8) [26]. When the missing mortality counts from the ONS were incorporated into the PHE-supplied data (PHE/ONS data), as shown in Additional file 1, the lifetime risk of diagnosis was 13.4 % (95 % CI, 13.3–13.5 %), or 1 in 8.

**Table 4:** Comparison of lifetime risk of prostate cancer diagnosis calculations in England/UK, by source

Source	Period	Lifetime risk	
		% (95 % CI)	Odds
CRUK ^a^	2010	13.2	1 in 8
ONS	2008–2010	13.1 (13.0–13.2)	1 in 8
NCDR	2008–2010	14.8 (14.7–14.9)	1 in 7
PHE/ONS ^b^	2008–2010	13.4 (13.3–13.5)	1 in 8

CRUK, Cancer Research UK; NCDR, National Cancer Data Repository; ONS, Office for National Statistics; PHE, Public Health England

^a^ CRUK calculation is based on data for the UK

^b^ PHE incidence and ONS mortality

Note: The analyses performed above on ONS and PHE data use the overall population estimates for 2008, 2009, and 2010 from ONS. These are slightly different from the overall numbers of the population estimates by ethnicity used in the main analyses

Table produced with data from [26, 33–35]

### Assigning an ethnicity to records with ‘unknown’ ethnicity

Missing ethnicity information can arise when there is no HES record matching a cancer registration or death certificate or no ethnic group recorded in HES. A large proportion of the data provided by PHE were categorized as having ‘unknown’ ethnicity, particularly in the NCDR prostate cancer incidence data (25.8 %; Table 5). Many prostate cancer incident cases do not require hospitalization. In 2012, only 12 % of men with prostate cancer had a radical prostatectomy (PHE, 2014, data by request) during which they would have been classed as an in-patient. Most others are treated as out-patients or in primary care where their ethnicity is infrequently documented. However, by the time of a man’s death, he is much more likely to have required hospitalization, which accounts for the lower proportion of missing ethnicity information in the mortality data (4.6 %; Table 5).

**Table 5:** Missing ethnicity information in the prostate cancer incidence/mortality and all-cause mortality data supplied by PHE

	Prostate cancer incidence		Prostate cancer mortality		All-cause mortality	
Period	Total, N	Unknown ethnicity, n (%)	Total, N	Unknown ethnicity, n (%)	Total, N	Unknown ethnicity, n (%)
2008	32,186	7,539 (23.4)	7,798	464 (6.0)	187,747	15,009 (8.0)
2009	35,243	8,822 (25.0)	8,174	391 (4.8)	185,127	11,348 (6.1)
2010	34,823	9,993 (28.7)	8,391	261 (3.1)	185,959	8,482 (4.6)
2008-2010	102,252	26,354 (25.8)	24,363	1,116 (4.6)	558,833	34,839 (6.2)

Table produced with data from [39]

To address the problem of missing ethnicity, three different methods were used to assign an ethnic group to the records with unknown ethnicity. The first method (the ‘All White’ method), at one extreme, assumed the ethnic group of all incident cases and deaths with an unknown ethnicity to be White. The second method (the ‘Proportionate’ method) assumed the ethnic groups of all incident cases and deaths with an unknown ethnicity were missing entirely at random and therefore would have the same distribution as the known cases and deaths. The third method (the ‘Increased Minority’ method), at the other extreme, assumed the ethnic group of incident cases and deaths with unknown ethnicity were more likely to be from a non-White ethnic group than in the ‘Proportionate’ method. Therefore, incidence cases and deaths with unknown ethnicity were assigned to the non-White ethnic groups by a further 50 % than the ‘Proportionate’ method. It was deemed too extreme to assume that all the incident cases and deaths with unknown ethnicity were from non-White ethnic groups, as the number with unknown ethnicity were several times higher than the total number known to be from non-White ethnic groups (Table 3). These three methods of assigning ethnicity were consistent with those used in a previous analysis of cancer incidence and survival by major ethnic group in England [13].

### Final datasets and ‘best estimate’ of lifetime risk

The all ethnicity-combined lifetime risk calculations (Table 4) confirmed the need to account for the discrepancy in overall number of deaths between the PHE-supplied and the ONS data. Lifetime risk calculations, by ethnic group, were therefore conducted using two sets of mortality data: the PHE mortality data as supplied (PHE) and the PHE data with the additional ONS deaths included (PHE/ONS). This second dataset, however, further compounded the issue of unknown ethnicity as any additional mortality counts from ONS lacked an ethnic classification. Table 4 shows the effect that the different sources of data and methods of assigning ethnicity had on the number of prostate cancer incident cases, prostate cancer deaths, and all-cause deaths by ethnic group. Irrespective of the method used, the majority of the incident cases and deaths were assigned to the White category, as the majority of the population, and therefore also of the known cases, were White. When including the additional deaths from the ONS data (PHE/ONS), the number of all-cause deaths with unknown ethnicity increased from 34,839 (PHE) to 147,573 (PHE/ONS).

The results of all three methods, on both datasets, together with their CIs provided a robust and reliable range in the absence of complete data. The ‘best estimate’ of lifetime risk was calculated by using the most complete data (the NCDR data for incidence and PHE/ONS data for deaths) and the ‘Proportionate Method’, as there was no known ethnic bias in the collection or linking of ethnicity data. Both the best estimates and the ranges, incorporating all assumptions and their CIs, are therefore presented throughout this study.

Although this study is based on data from England only (due to difficulties in sourcing data for Scotland, Wales, and Northern Ireland), the results can be assumed to be representative of men across the United Kingdom, as in the PROCESS study [14].

The full dataset of raw and manipulated data can be found in Additional file 2.

## Results

### All ethnicities

For all ethnicities combined, the lifetime risk of being diagnosed with prostate cancer in England in 2008–2010, using the most complete data available, was 13.4 % (1 in 8; 95 % CI, 13.3–13.5 %; Table 4). The lifetime risk of dying from prostate cancer in England has not previously been published. For all ethnicities combined, the lifetime risk of dying from prostate cancer in England in 2008–2010, using ONS mortality data, was 4.3 % (1 in 24; 95 % CI, 4.2–4.3 %; data not shown).

### By major ethnic group

**Fig. 1:**
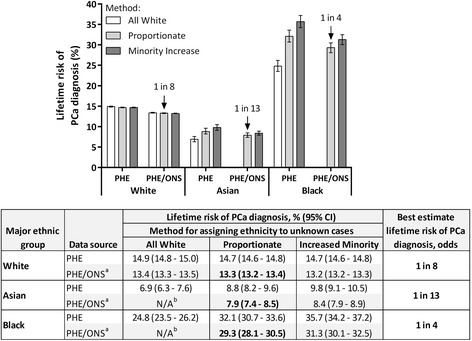
Lifetime risk of being diagnosed with prostate cancer by major ethnic group and methodology, England 2008–2010. ONS, Office for National Statistics; PCa, Prostate cancer; PHE, Public Health England. Best estimate lifetime risk (% and odds) are highlighted in bold. ^a^ PHE incidence and mortality data, with additional ONS mortality counts. ^b^ For non-White ethnic groups, the All White method does not apply to the PHE/ONS data, as any additional deaths from the ONS data would be coded as White and therefore not impact the analysis of the non-White group

**Fig. 2:**
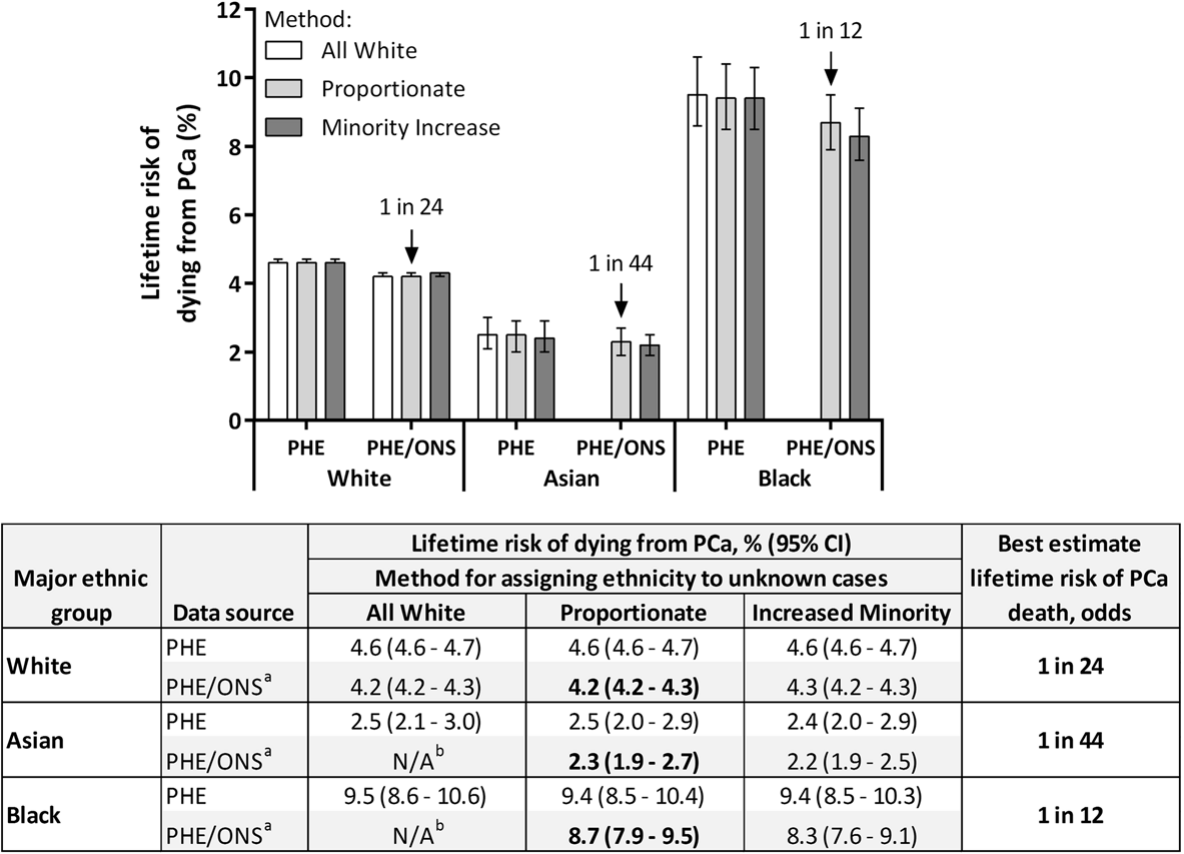
Lifetime risk of dying from prostate cancer by major ethnic group and methodology, England 2008–2010. ONS, Office for National Statistics; PCa, Prostate cancer; PHE, Public Health England. Best estimate lifetime risk (% and odds) are highlighted in bold. ^a^ PHE incidence and mortality data, with additional ONS mortality counts. ^b^ For non-White ethnic groups, the All White method does not apply to the PHE/ONS data, as any additional deaths from the ONS data would be coded as White and therefore not impact the analysis of the non-White groups

Figures 1 and 2 show the lifetime risk of being diagnosed with, and dying from, prostate cancer, respectively, for the three different methods of assigning an ethnic group to incident cases and deaths with unknown ethnicity, within each of the two datasets. The width of the CIs within each ethnic group is inversely proportionate to the size of the population; with White men being the largest group (22.2 million) and having the smallest CIs and Black men being the smallest group (0.8 million) and having the largest CIs (Table 1; Figs. 1 and 2). The ranges presented below combine the results of all analyses and their CIs and therefore combine uncertainty about the unknown ethnicities (as measured by the different analyses) and statistical uncertainty (as measured by the CI around each estimate).

The lifetime risk of being diagnosed with prostate cancer for White men for the period 2008–2010 ranged from 13.2 % to 15.0 %, with a best estimate of 13.3 % (1 in 8; Fig. 1). The results were consistent across the three methods of assigning unknown ethnicity, with the width of the range being primarily due to the source of mortality data. The lifetime risk of dying from prostate cancer for White men ranged from 4.2 % to 4.7 %, with a best estimate of 4.2 % (1 in 24; Fig. 2). Both the lifetime risk of being diagnosed with, and dying from, prostate cancer in White men were similar to all ethnicities combined. This was to be expected, as 87 % of all men in England in 2008–2010 were estimated to be White (Table 2).

The lifetime risk of being diagnosed with prostate cancer for Asian men for the period 2008–2010 ranged from 6.3 % to 10.5 %, with a best estimate of 7.9 % (1 in 13; Fig. 1). This was the lowest of the three major ethnic groups analyzed. The lifetime risk of dying from prostate cancer for Asian men ranged from 1.9 % to 3.0 %, with a best estimate of 2.3 % (1 in 44; Fig. 2). This was also the lowest of the three major ethnic groups analyzed. Asian men were at a significantly lower risk of being diagnosed with, and dying from, prostate cancer in their lifetime compared to White men.

The lifetime risk of being diagnosed with prostate cancer for Black men for the period 2008–2010 ranged from 23.5 % to 37.2 %, with a best estimate of 29.3 % (1 in 4; Fig. 1). This was the highest of the three major ethnic groups analysed. The range was wider than other ethnic groups due to Black men having the highest prostate cancer incidence rate (179.4 per 100,000; PHE, 2014, data on request) and therefore the lifetime risk calculation was more strongly influenced according to which method was used to assigning an ethnicity to unknown cases. The lifetime risk of dying from prostate cancer for Black men ranged from 7.6 % to 10.6 %, with a best estimate of 8.7 % (1 in 12; Fig. 2). This was also the highest of the three major ethnic groups analyzed.

## Discussion

This is the first study to break down the lifetime risk of being diagnosed with, and dying from, prostate cancer in England by major ethnic group. We have shown that Black men are at double the lifetime risk of both being diagnosed with and dying from prostate cancer, compared to White men in England. Asian men are at just over half the lifetime risk of both being diagnosed with and dying from prostate cancer, compared to White men in England.

When comparing the lifetime risk of dying from prostate cancer with the lifetime risk of being diagnosed with prostate cancer within each ethnic group, the ratios were very similar and all close to one third (Figs. 1 and 2). This shows that once a man has been diagnosed with prostate cancer, he has a one third chance of dying from the disease, independent of his ethnicity. This could be interpreted as an indication that the disease is no more aggressive in any one ethnic group and/or that there is no bias in detection or treatment between ethnic groups. Nonetheless, proportionally more Black men are dying from prostate cancer in England.

### Limitations of this study

Firstly, the analyses performed in this study were based on a number of assumptions and considerations, most of which were required to address the lack of available data by ethnicity. This highlights the urgent need for more routine collection of data that captures ethnicity to ensure that researchers can more accurately evaluate whether inequalities exist. For records with complete ethnicity data, it is worth noting that this is based on self-reported ethnicity. Secondly, this study does not provide any information on men of mixed ethnicity since the minor ethnic groups within the major ethnic group ‘Mixed’ include a wide range of mixed ethnicities, making it difficult to attribute any potential differences in risk to one particular ethnicity. Finally, since the mortality data in this study corresponded to men who died from prostate cancer between 2008 and 2010, the majority of these deaths may have been from prostate cancers diagnosed before 2008. Therefore, the ratio of prostate cancer deaths to diagnoses should be interpreted with caution.

## Conclusions

The NCDR-HES and HES-ONS linked datasets have enabled this new analysis of prostate cancer data by ethnicity. Our findings are of importance to primary and secondary healthcare professionals working within Black communities and Black men themselves. The importance of this data has already been recognised by NHS England in their ‘Be Clear on Cancer – Prostate Cancer’ campaign, piloted in London in 2014, of which the headline message was “I didn’t know 1 in 4 Black men get prostate cancer. Did you?” [44].

Our finding that Black men are at double the lifetime risk of being diagnosed with prostate cancer in England, compared to White men, provides Black men with important and useful information. The first step towards a diagnosis of prostate cancer is often a PSA blood test and, due to the high likelihood of false positive or false negative results, information about prostate cancer risk is an important factor for men when deciding whether or not to have a PSA test. To date, relative risk (to that of White men) has been used to communicate to Black men their increased risk of being diagnosed with prostate cancer. However, the data in this study provides, for the first time, prostate cancer lifetime risk data tailored by ethnic group. This tailored information in the form of an absolute risk figure is important for targeted awareness-raising amongst Black men of their higher than average risk. Recent recommendations on communicating risk suggest that absolute risk, rather than relative risk, can help improve understanding and decision making [45]. We therefore believe the lifetime risk figures in this study will help Black men better understand their risk of developing prostate cancer and make an informed decision about whether or not to have a PSA test.

It is important to remember that every individual’s risk is different and will vary based on a combination of different factors in addition to ethnicity, such as age, family history of prostate cancer, and body weight. However, these new figures on lifetime risk of diagnosis by ethnic group are an important tool for discussion of prostate cancer risk with men. Additionally, the new figures on the increased lifetime risk of dying from prostate cancer may provide the rationale for a future trial of a targeted prostate cancer screening programme in Black men. Although there is not yet evidence that the benefits of screening an entire population of men for prostate cancer outweigh the risks [46, 47], we need to understand whether there would be an improvement in the benefit–risk ratio for screening targeted populations at higher than average risk of developing and dying from prostate cancer. It is also important to remember that lifetime risk calculations are based on current incidence and mortality rates. Prostate cancer incidence rates have been rising since around the year 2000 [48]; if this trend continues, then younger generations may be at a higher lifetime risk of being diagnosed with prostate cancer than the current estimate.

Future research needs to address what lies behind the variations in prostate cancer risk based on ethnicity, shown in this study. Ongoing research into genetic biomarkers may begin to account for some of the difference in risk. Further data collection is required on PSA testing rates in primary care, broken down by ethnic group, to determine whether Black men are more likely to be diagnosed with aggressive disease. Most health databases have the facility to record ethnicity but there can be a reluctance to complete these data. The limitations of this study support continued calls for better collection of ethnicity data in order to better understand differences based on ethnicity and to ultimately ensure all men receive the best level of tailored prostate cancer information, treatment, and care.

## Additional files

Additional file 1:
**Sources of data used.** This flow chart shows the raw data sources used to gather statistics on prostate cancer incidence, prostate cancer mortality, all-cause mortality and population estimates, available where possible by ethnic group, and then how these data were subsequently combined in order to produce the final datasets (shown in grey boxes) required in order to calculate the lifetime risk of being diagnosed with, and dying from, prostate cancer by major ethnic group. Additional file 2:
**Raw and manipulated dataset.**

## Abbreviations

CI: Confidence interval
HES: Hospital Episode Statistics
NCDR: National Cancer Data Repository
NHS: National Health Service
ONS: Office for National Statistics
PHE: Public Health England
PROCESS: Prostate Cancer in Ethnic Subgroups
PSA: Prostate-specific antigen
