# Supplementary material for: Lifetime risk of being diagnosed with, or dying from, prostate cancer by major ethnic group in England 2008–2010
Source: BMC Med. 2015 Jul 30;13:171. doi: 10.1186/s12916-015-0405-5 (PMC4520076; doi:10.1186/s12916-015-0405-5)
Supplement: Additional file 1: — Sources of data used. This flow chart shows the raw data sources used to gather statistics on prostate cancer incidence, prostate cancer mortality, all-cause mortality and population estimates, available where possible by ethnic group, and then how these data were subsequently combined in order to produce the final datasets (shown in grey boxes) required in order to calculate the lifetime risk of being diagnosed with, and dying from, prostate cancer by major ethnic group. [file 12916_2015_405_MOESM1_ESM.pptx]

## Slide 1
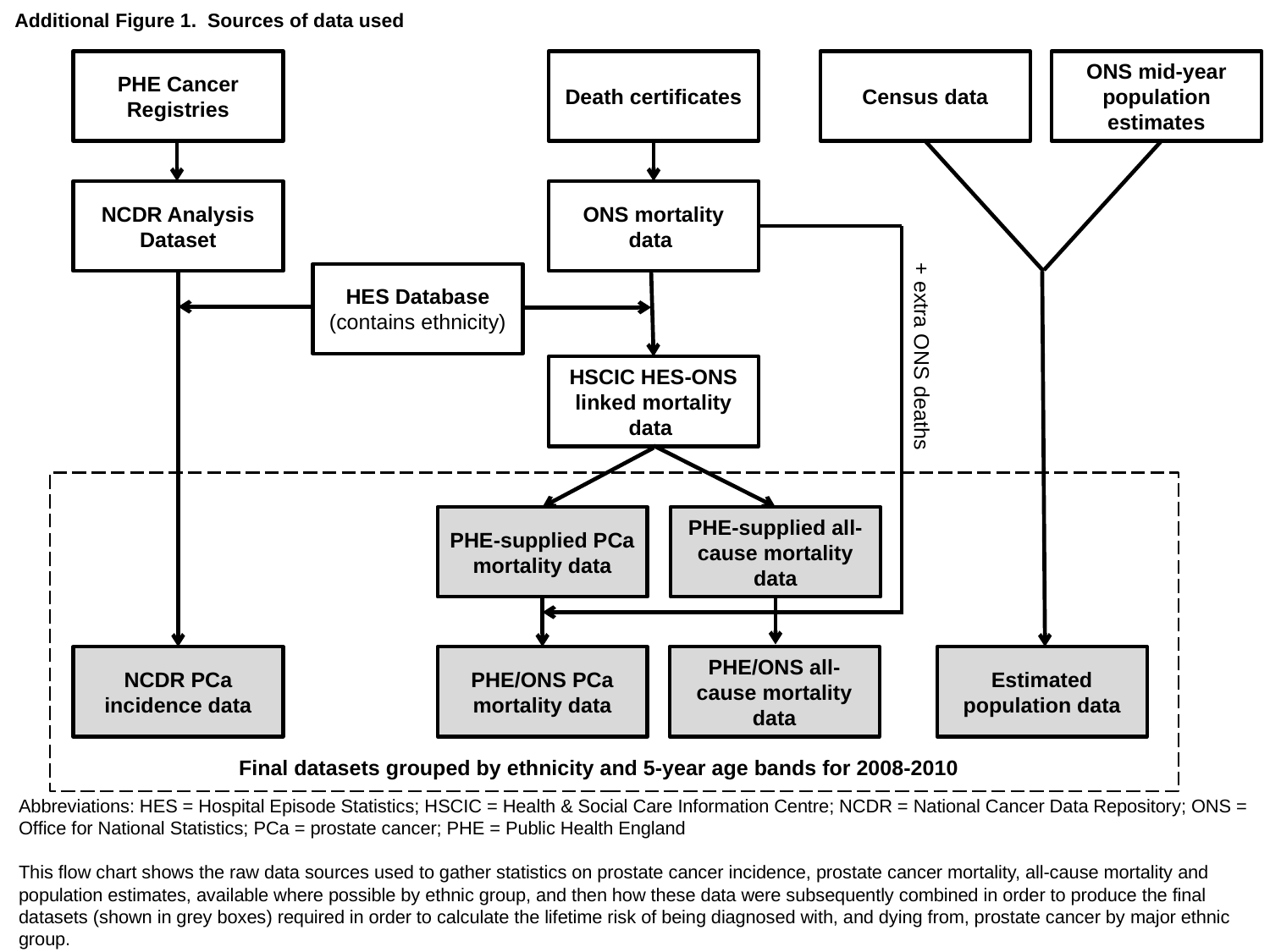

Additional Figure 1. Sources of data used
PHE Cancer Registries
Death certificates
Census data
ONS mid-year population estimates
NCDR Analysis Dataset
ONS mortality data
HES Database
(contains ethnicity)
+ extra ONS deaths
HSCIC HES-ONS linked mortality data
PHE-supplied PCa mortality data
PHE-supplied all-cause mortality data
NCDR PCa incidence data
PHE/ONS PCa mortality data
PHE/ONS all-cause mortality data
Estimated population data
Final datasets grouped by ethnicity and 5-year age bands for 2008-2010
Abbreviations: HES = Hospital Episode Statistics; HSCIC = Health & Social Care Information Centre; NCDR = National Cancer Data Repository; ONS = Office for National Statistics; PCa = prostate cancer; PHE = Public Health England
This flow chart shows the raw data sources used to gather statistics on prostate cancer incidence, prostate cancer mortality, all-cause mortality and population estimates, available where possible by ethnic group, and then how these data were subsequently combined in order to produce the final datasets (shown in grey boxes) required in order to calculate the lifetime risk of being diagnosed with, and dying from, prostate cancer by major ethnic group.
